# A social network intervention to improve connectivity and burnout among psychiatry residents in an academic institution: a quasi-experimental study

**DOI:** 10.1186/s12909-022-03440-5

**Published:** 2022-05-13

**Authors:** Ardavan Mohammad Aghaei, Vandad Sharifi, Maryam Tabatabaee, Fattaneh Abdi-Masouleh, Reza Yousefi Nooraie

**Affiliations:** 1 Department of Psychiatry, School of Medicine, Tehran University of Medical Sciences, Roozbeh Hospital, South Kargar Avenue, 13337-15914 Tehran, Iran; 2 Department of Public Health Sciences, School of Medicine and Dentistry, University of Rochester, Rochester, USA

**Keywords:** Burnout, Medical education, Residency program, Hospital education environment, Social network analysis

## Abstract

**Background:**

Burnout is common among residents, which could be associated with their professional network characteristics. This study aimed to assess the social networks of psychiatry residents and develop an intervention to improve their network characteristics, burnout, and perception of the educational environment.

**Methods:**

We recruited a cohort of 17 PGY-2 residents and assessed their social networks, burnout, and perception of the educational environment. After the baseline survey, we held a focus group with PGY-2 residents to discuss the results, their network characteristics, and interventions that can improve their relationships. The PGY-2 residents indicated that offering extracurricular opportunities to facilitate friendly interactions among the residents and faculty members would be the most feasible and acceptable intervention. Therefore, four “interest groups” for extracurricular activities were established. Residents and faculty members were invited to participate in interest groups to improve the network characteristics. Some PGY-2 residents and faculty members agreed to moderate interest group sessions (active members).

**Results:**

After the intervention, active residents improved significantly in the perceived personal accomplishment subscale of the burnout inventory and their perception of the educational environment. Active faculty members also had a significant increase in their relationships with PGY-2 residents in one domain of social networks.

**Conclusions:**

Enhancing relationships between residents and faculty members through participatory intervention and extracurricular activities can improve faculty-resident connectivity and residents’ perception of personal accomplishment and educational environment quality.

**Supplementary information:**

The online version contains supplementary material available at 10.1186/s12909-022-03440-5.

## Introduction

Teaching hospitals are multi-purpose communities with different, and often conflicting, aims: providing quality care, conducting health research, and training competent physicians. In this environment, the well-being, burnout, and academic performance of residents, given their dual responsibility both as a physician and a trainee, is a matter of concern [1]. These complexities, in conjunction with long shifting hours and low payment in some teaching hospitals, predispose residents to burnout. Existing literature suggests that burnout is quite common among residents [2, 3], and leads to clinical errors, poor rapport with the patients, and lower academic performance [4]. Given the importance of the issue, several studies have tried different intervention approaches to reduce residents’ burnout which demonstrated different effectiveness [5].

Social network analysis (SNA) research has investigated the effect of social contagion and influence on different aspects of life [6]. Personal characteristics such as weight [7], behaviors [8], or even intrapsychic experiences such as happiness [9], are correlated, and in some studies causally related, to one’s network characteristics in various domains of social connectedness. SNA is a collection of methods that investigate relationships, interactions, and social structures. SNA could visualize interactions between actors in a social network and help recognize groups, influential actors, and relationship patterns. Alongside mapping the network, SNA could quantify network parameters such as the number of interactions each person receives (indegree) or contributes to (outdegree), and the role and position of an actor in the network (centrality measures). SNA not only helps to understand the communities but also provides the researchers with various tools to design interventions to change them [6, 10–12].

In health system research, SNA has indicated that network characteristics of the staff are associated with burnout and job satisfaction [13, 14]. In educational sciences, SNA has given the researchers valuable insights into how dissimilarities in students’ social network properties influence their academic performance, well-being, and perception of accomplishment [15, 16]. Some consider social networks as an important part of the hidden curriculum [17–19]. However, despite the growing utilization of this method in other fields, SNA studies in the field of health education are rare [20].

In this study, we aimed to elicit the social network of a class of residents in an academic psychiatric hospital (Roozbeh) in Tehran, Iran. We also intended to develop and implement a participatory intervention informed by network analysis to improve residents’ burnout and their perception of the educational environment.

## Methods

### Context and participants

The physician community of the hospital consists of faculty members and psychiatry residents. Between 16 and 20 physicians start their psychiatry residency program at the hospital each year. The psychiatry residency is a four-year, competency-based program with a combination of supervised clinical experiences in different settings and a comprehensive didactic program. The pillars of the residency curriculum are quite similar to most North American programs. The resident-faculty relationship is usually limited to clinical settings, seminars, journal clubs, and other didactic sessions.

### Baseline and follow up surveys

The baseline survey took place in December 2018. At the time, the hospital physician network consisted of 17 PGY-2 residents (participants), 34 PGY-3 and PGY-4 residents, and 47 faculty members. The follow-up survey took place in January 2020. The PGY-2 residents completed the study questionnaire at baseline and follow-up, which included the social networks questionnaire, the Maslach Burnout Inventory (MBI), and the Postgraduate Hospital Educational Environment Measure (PHEEM).

To assess the social networks and develop the intervention, we approached PGY-2 residents because they had already spent a year together building some relationships and had enough time ahead to get engaged in an intervention. PGY-2 residents were asked to nominate other residents and faculty members from a roster of hospital physicians with whom they had a social relationship in three different domains (clinical advice, educational advice, and personal support) in the previous month.

The survey also included the Maslach Burnout Inventory (MBI) [21] and the Postgraduate Hospital Educational Environment Measure (PHEEM) [22]. The MBI measures burnout in three dimensions: emotional exhaustion, depersonalization, and perceived sense of personal accomplishment. The PHEEM is a 40-item questionnaire designed specifically for assessing residents’ perceptions of the hospitals’ teaching environment. The reliability and validity of the Persian version of both instruments had been evaluated previously [23, 24].

### Intervention design and implementation

The intervention was designed in a participatory manner. After the baseline survey and data extraction, we held a focus group with the PGY-2 residents. Fifteen out of 17 PGY-2 residents attended the focus group. The network maps and a brief report on burnout levels and PHEEM scores were presented to the PGY-2 residents. The PGY-2 residents were asked to brainstorm about the networks and interventions that can improve their relationship and perceived support.

According to the PGY-2 residents, their networks with each other were acceptably dense and balanced and needed no intervention. They also felt they had enough access to senior residents, and the amount of interaction was satisfactory. However, the PGY-2 residents felt a need for more interaction with faculty members, especially in a more informal manner. After more discussion about network characteristics, PGY-2 residents extensively reviewed potential solutions. PGY-2 residents indicated that offering extracurricular opportunities to facilitate friendly interactions among residents and faculty members would be the most feasible and acceptable intervention. Therefore, four “interest groups” focusing on extracurricular activities (psychiatry and literature, psychiatry and cinema, psychiatry and philosophy, and hiking) were established, and residents and faculty members were invited to participate. There was no limitation on the number of interest groups that residents and faculty members could attend.

To ensure appropriate management of the interest groups, one or two volunteer PGY-2 residents and one or two faculty members were assigned to moderate each interest group. This group will be called “active PGY-2 residents” and “active faculty members”, collectively called “active members”. The non-active members were also invited to and could attend all interest groups’ meetings; the only difference was involvement in interest groups’ moderation. Seven PGY-2 residents and six faculty members volunteered to serve as active members. Each interest group was expected to hold one meeting per month. The investigators did not intervene in the interest group’s management. During the seven-month intervention period, three interest groups held six meetings each, and one held five meetings.

### Analysis

The network maps and indicators were created using UCINET software [25]. Two groups of networks were considered in the analysis: the relationship of PGY-2 residents within their cohort (one-mode networks), and the relationship of PGY-2 residents with faculty members (two-mode networks). The intervention aimed to improve connectivity between PGY-2 residents and faculty members; therefore, we excluded PGY-2 residents’ relationships with senior residents from the analysis.

We calculated the following indicators of the network structure: density (the number of existing ties to total possible ties), reciprocity (the number of bidirectional relationships to total existing relationships), and indegree centralization (ranges between zero and one, which measures the extent ties are focused on one or a few people). The significance of network structure differences between baseline and follow-up was calculated via bootstrap t test with 5000 samples using UCINET software.

We developed mixed-effect linear regression models with restricted maximum likelihood ratios to assess the changes in outcomes (the indegree of each member in networks, PHEEM, and MBI dimensions scores). For each model, the predictors included the “active vs. non-active members” and a variable indicating “baseline vs. follow-up” and their interaction. In models assessing PGY-2 residents’ changes, the PGY-2 resident’s ID number and in the models assessing faculty members’ changes, the faculty member’s ID number, were considered as the random effect. The models were developed by the “lme4” and “lmeTest” packages of R software version 4.03 [26, 27].

## Results

### Networks of PGY-2 residents

All 17 PGY-2 residents agreed to participate in the study (14 females and three males). The median age of PGY-2 residents was 32 years. One was excluded from the study because of failing the annual evaluation exam resulting in a different educational trajectory at follow-up.

**Fig. 1:**
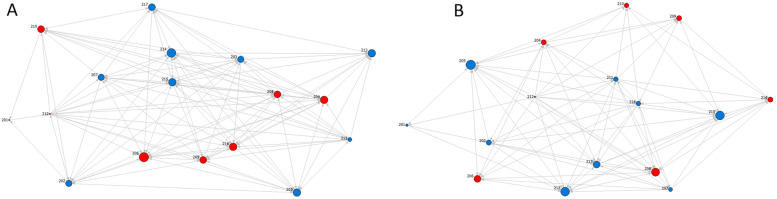
The PGY-2 residents’ networks(one-mode) at baseline (**A**) and follow-up (**B**). Each node represents one PGY-2 resident, and each arrow is a nomination (personal support or clinical or educational advice). The direction of the arrow is from the nominator to the nominee. The size of each node is proportionate to its indegree. The red nodes are PGY-2 residents who volunteered to act as interest group moderators (active PGY-2 residents)

Figure 1 shows the PGY-2 residents’ network maps based on all relationship types at baseline and follow-up. Relationship-specific network maps are provided in Additional file 1: supplementary figure S1-S3. The maps are one-mode networks that show only connections among the PGY-2 residents’ cohort. As shown in Fig. 1; Table 1, all three network types were relatively well connected and dense at baseline. The personal support network displayed the largest density (0.45), and reciprocity (0.28). Indegree centralizations in all three network types were small, which implies that the connections were evenly distributed and not monopolized by a few PGY-2 residents.

At follow-up, the densities slightly decreased, which was statistically significant in the personal support network. In all three networks, we observed a decrease in reciprocity and a mild elevation in indegree centralization, implying a tendency towards hierarchy.

**Table 1:** The structural indicators of different PGY-2 residents’ (one-mode) networks at baseline and follow-up

	Personal support network		Clinical advice network		Educational advice network	
	Baseline	Follow-up	Baseline	Follow-up	Baseline	Follow-up
Average degree	7.18	5.30	4.05	3.00	3.64	3.13
Density	0.45	0.35^*^	0.25	0.20	0.23	0.21
Reciprocity	0.28	0.21	0.15	0.09	0.13	0.06
Indegree centralization	0.19	0.26	0.19	0.21	0.29	0.35

* Bootstrap t test to compare densities (5000 samples): *p* < 0.05

### Participation of active and non-active members

The difference in interest group attendance between active and non-active members was significant for both PGY-2 residents and faculty members (P values < 0.01). Non-active PGY-2 residents, on average, attended 1.3 sessions, while active PGY-2 residents attended eight sessions. Similarly, the average attendance for non-active faculty members was 0.7 sessions, and for active faculty members was 6.8 sessions.

### Active and non-active members at baseline

Active PGY-2 residents did not differ from other PGY-2 residents in any network properties at baseline (Table 2). However, they had significantly higher scores in depersonalization and lower scores of perceived personal accomplishments in burnout sub-scales.

Active faculty members had significantly more relationships with PGY-2 residents than their non-active peers in clinical advice and educational advice networks at baseline (Table 2).

**Table 2:** The intercept and coefficients of linear mixed-effect regressions for different outcomes and their statistical significance

	PGY-2 residents (one-mode^a^) Networks Normalized Indegrees			Faculty members (two-mode^b^) Networks Normalized Indegrees			Maslach Burnout Inventory			PHEEM Score
	Personal Support	Clinical Advice	Educational Advice	Personal Support	Clinical Advice	Educational Advice	Emotional Exhaustion	Depersonalization	Personal Accomplishment	
Baseline: Non-active members (intercept)	0.450	0.231	0.206	0.032	0.051	0.039	10.90	2.20	38.90	90.14
Baseline: Difference of active^c^ vs. non-active members	-0.003	0.054	0.052	0.065	0.086*	0.098**	-0.04	3.37*	-4.90*	-1.71
Non-active members: Difference of follow-up vs. baseline	-0.067	-0.034	0.017	-0.009	0.004	-<0.000	11.69**	0.78	-4.56*	-8.44*
Active members: Difference of follow-up vs. baseline	-0.122**	-0.085*	-0.077	0.068**	-0.033	-0.012	5.85	< 0.01	5.14*	12.42**
Difference in differences: Differential changes in active vs. non-active over time	-0.054	-0.051	-0.095	0.078**	-0.037	-0.011	-5.83	-0.78	9.71**	20.86**

Definitions: Normalized indegree = Total number of nominations an individual receives in a network; reported as a fraction of total possible nominations.

* = Statistically significant with *P* < 0.05

** = Statistically significant with *P* < 0.01

^a^ Social networks that both nominators and nominees are PGY-2 residents

^b^ Social networks that nominators are PGY-2 residents, but nominees are faculty members (who did not participate in the survey themselves)

^c^ PGY-2 residents and faculty members who acted as moderators of intervention (interest groups)

### Active and non-active members over time

Active PGY-2 residents reported a significant increase in their perceived personal accomplishment in the MBI and PHEEM scores compared to their baseline (Table 2). While non-active PGY-2 residents reported significantly higher emotional exhaustion, lower perceived personal accomplishment, and lower PHEEM scores compared to their baseline. The differential improvement of active vs. non-active PGY-2 residents with respect to PHEEM scores and personal accomplishment was also statistically significant. Active PGY-2 residents had a decrease in indegree in the one-mode networks (connection within their cohort), which was significant in personal support and clinical advice networks, while the decrease in indegree of non-active PGY-2 residents was not statistically significant.

Active faculty members had significantly more relationships with PGY-2 residents in the personal support network compared to baseline. No significant change was observed in the network indicators of non-active faculty members. The differential increase of active faculty members’ relationships vs. non-active faculty members in the personal network over time was statistically significant.

## Discussion

To the best of our knowledge, this study is the first to implement an intervention to improve residents’ burnout and their perception of the educational environment informed by SNA. We examined the social network of PGY-2 psychiatry residents in a teaching hospital, which showed an evenly distributed network between PGY-2 residents and limited interactions with faculty members. In the focus group discussion, the PGY-2 residents expressed their desire to improve their relationships with the faculty members. Our intervention successfully engaged a group of PGY-2 residents and faculty members as champions and group moderators of interest groups (active members). After seven months of the intervention, we found that the intervention significantly increased the relationships of active faculty members with PGY-2 residents and improved burnout and perception of the educational environment in active PGY-2 residents.

### Impact on burnout and educational environment perception

The results show that the intervention was particularly beneficial for active PGY-2 residents, who had an improved perception of personal accomplishment at follow-up. This finding is in concordance with a published cross-sectional study indicating that out of burnout sub-scales, only perception of personal accomplishment is associated with social network position [28]. Interestingly, a systematic review that studied interventions addressing burnout among residents concluded that interventions by approaches other than SNA would improve emotional exhaustion and, to some degree, depersonalization sub-scales. The reported interventions emphasized workload reduction and self-care behaviors [5]. It can be concluded that interventions that aim to improve connectedness may address an aspect of burnout syndrome that otherwise remains untouched- the perception of personal accomplishment, which needs to be confirmed by further studies.

Active PGY-2 residents also reported an improved perception of the educational environment. This finding is in line with existing literature. We know that while faculty-student relationships affect students’ performance, they are usually scarce and limited to academic interactions [29]. Studies have shown that providing opportunities for interaction between students and faculty members facilitates the development of chemistry and mentor-mentee relationships [30–33]. The faculty-resident relationship is identified as an important determinant of residents’ well-being [34]; and similarly, having seniors in the social network will help students’ academic performance [35].

Unlike their peers, non-active PGY-2 residents reported significantly higher emotional exhaustion and a declining perception of personal accomplishment and of the educational environment quality. During the intervention period, several stressful events happened in the country, such as a serious economic crisis that posed stress to the public and might have contributed to this higher burnout in non-active PGY-2 residents. But it is also possible that the intervention design per se could be partly responsible for the unfavorable outcome observed in non-active PGY-2 residents. A similar pattern was seen in another study aimed to improve self-care behavior in residents by an intervention involving some residents and informing other residents about their progress via email, hypothesizing that the emails would improve other residents’ self-care by social contagion. The authors found that other residents who were not directly involved in the intervention neither welcomed nor benefited from emails [36]. The researchers attributed this effect to the “fear of missing out” [37] and the “feeling of inferiority when comparing oneself to others” [38].

### Impact on the networks

The intervention successfully increased the relationships of active faculty members with PGY-2 residents. At follow-up, active faculty members had a significant increase in their relationships with PGY-2 residents at the personal support network. Although, this improvement did not extend to the non-active faculty members’ relationships. It is not unusual in social network studies that interventions impact actors differently. The concept of preferential attachment suggests that the actors with more social ties have more chance of developing new ties due to intervention, a phenomenon that results in a “rich get richer” pattern after network interventions [39]. This implies that in any network intervention, special attention should be paid to involving actors in the network periphery.

At the time of preparing this manuscript (one year after follow-up surveys), three out of four interest groups are still running while investigators had no contribution to it. The interest groups survived the COVID-19 pandemic by using online interfaces. This indicates that the effects of the intervention may have gone beyond what we have reported here.

### Limitations

We faced some limitations in our study, namely, the quasi-experimental design without a control group which was implemented in a real-life environment. There were numerous confounding variables that we were unable to control. Therefore, the conclusions we made in this manuscript are merely hypotheses in need of further confirmation in controlled interventional studies. Our small sample size limits the statistical power of the study. Furthermore, we did not assess the quality (frequency and intensity) of relationships, which may have changed due to intervention.

## Conclusions

Enhancing relationships between residents and faculty members through participatory intervention and extracurricular activities can improve faculty-resident connectivity and residents’ perception of personal accomplishment and of the educational environment quality.

## Supplementary Information

**Additional file 1.**

## Data Availability

The datasets generated and/or analyzed during the current study are not publicly available due to privacy and ethical restrictions but are available from the corresponding author upon reasonable request.

## Abbreviations

SNA: social network analysis
PGY: postgraduate year
MBI: Maslach Burnout Inventory
PHEEM: Postgraduate Hospital Educational Environment Measure
