# Supplementary material for: A social network intervention to improve connectivity and burnout among psychiatry residents in an academic institution: a quasi-experimental study
Source: BMC Med Educ. 2022 May 13;22:367. doi: 10.1186/s12909-022-03440-5 (PMC9098378; doi:10.1186/s12909-022-03440-5)
Supplement: Supplementary file 1 — Additional file 1. [file 12909_2022_3440_MOESM1_ESM.docx]

**Supplementary figures**


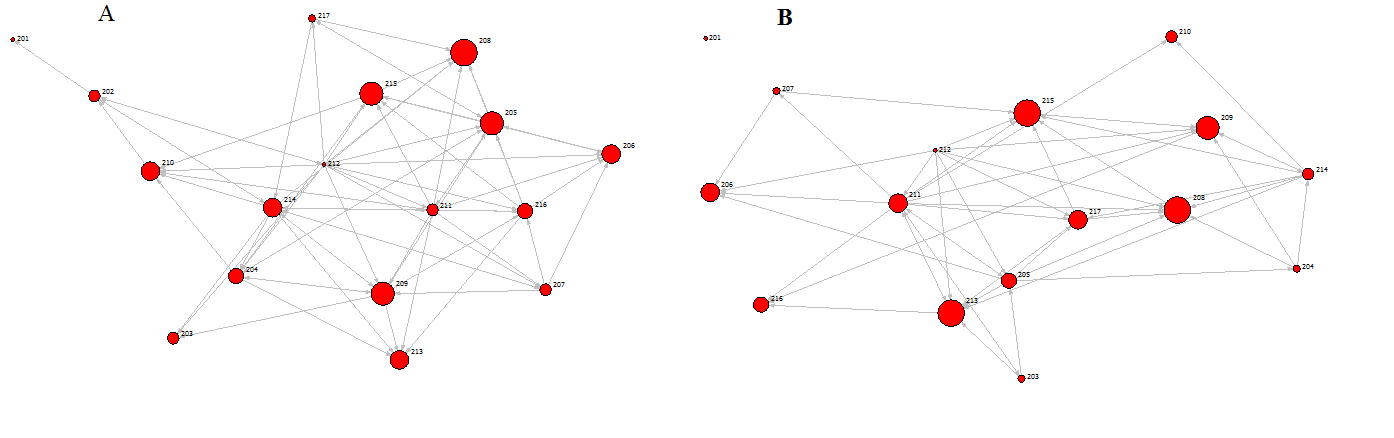


Figure S1: The PGY-2 residents’ clinical advice networks(one-mode) at baseline (A) and follow-up (B). Each node represents one PGY-2 resident, and each arrow is a nomination. The direction of the arrow is from the nominator to the nominee. The size of each node is proportionate to its indegree.


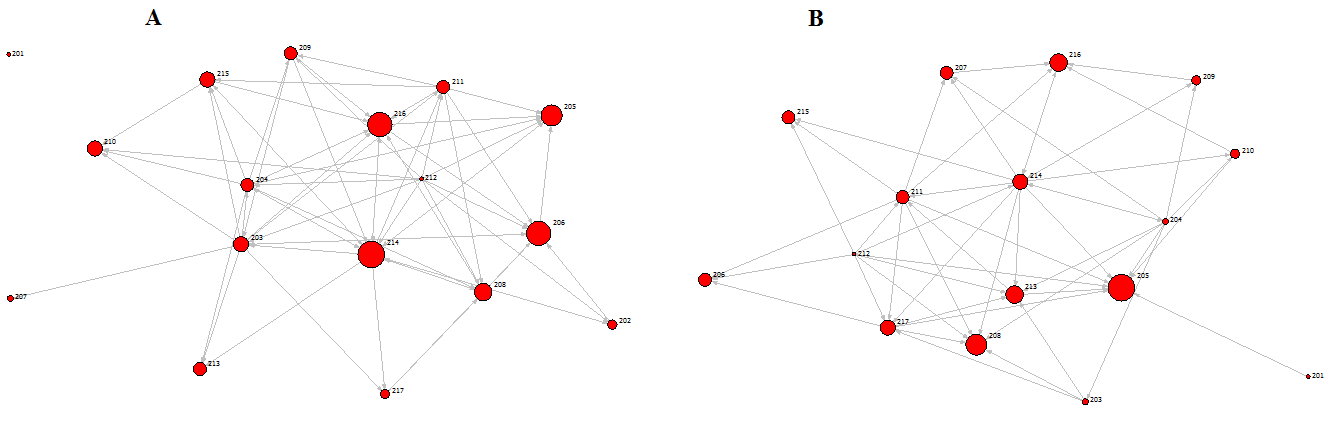


Figure S2: The PGY-2 residents’ educational advice networks(one-mode) at baseline (A) and follow-up (B). Each node represents one PGY-2 resident, and each arrow is a nomination. The direction of the arrow is from the nominator to the nominee. The size of each node is proportionate to its indegree.


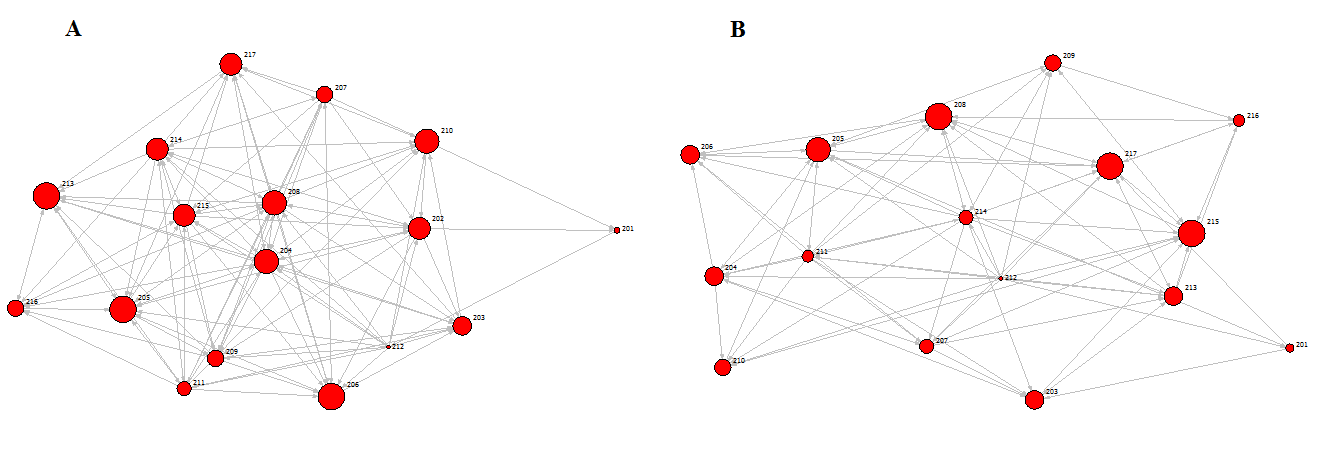


Figure S3: The PGY-2 residents’ personal support networks(one-mode) at baseline (A) and follow-up (B). Each node represents one PGY-2 resident, and each arrow is a nomination. The direction of the arrow is from the nominator to the nominee. The size of each node is proportionate to its indegree.
